# Introducing physical exercise as a potential strategy in liver cancer prevention and development 

**Published:** 2021

**Authors:** Mona Zamanian-Azodi, Sakineh Khatoon Hajisayah, Mohhamadreza Razzaghi, Mostafa Rezaei-Tavirani

**Affiliations:** 1 *Proteomics Research Center, Shahid Beheshti University of Medical Sciences, Tehran, Iran*; 2 *Department of Basic Sciences, School of Rehabilitation Sciences, Shahid Beheshti University of Medical Sciences, Tehran, Iran*; 3 *Laser Application in Medical Sciences Research Center, Shahid Beheshti University of Medical Sciences, Tehran, Iran*; 4 *Proteomics Research Center, Faculty of Paramedical Sciences, Shahid Beheshti University of Medical Sciences, Tehran, Iran *

**Keywords:** Physical activity, Liver health, Protein-protein interaction network analysis, Gene ontology, Anticancer

## Abstract

**Aim:**

This study aimed to investigate the anticancer properties of physical activity by network analysis in trained rats.

**Background:**

Much evidence supports the benefits of physical activity, most of which are related to metabolism regulation and body health. Deeper investigation deals with other features of physical activity, such as its anticancer properties

**Methods:**

Protein-protein interaction network analysis was applied to investigate the proteome profile of livers of rats subjected to physical activity through bioinformatics. Twelve differentially expressed proteins were searched and analyzed by Cytoscape 3.7.2 and its plug-ins. The network was analyzed to identify hub-bottleneck nodes. An action map was constructed for the central proteins.

**Results:**

Among the queried proteins, Eno1 and Pgm1 were only assigned as hubs by Network Analzyer. Gpi, Pkm, Aldoa, and Aldoart2 were identified as central nodes among the first neighbors of network elements. Furthermore, the glycolytic, carbohydrate catabolic, and glucose metabolic processes are key elements that could be imperative in the mechanism of exercise in liver function. The anticancer properties of the central nodes were highlighted.

**Conclusion:**

The network findings indicate the anticancer properties of physical activity, which has also been supported by previous investigations.

## Introduction

 The impacts of physical fitness on different kinds of diseases and improvement of organ functions have been of interest to many researchers (1, 2). Health promotion of the liver as one the critical organs of the human body is essential, such that exercise training could be beneficial for this purpose (3). In general, exercise could modulate the functions of major organs such as the heart, brain, and liver (4-6). In addition, the role of exercise training in improving the outcomes of diseases such as non-alcoholic hepatic steatosis (6), fatty liver disease (2), neurodegenerative diseases (1), and advanced stages of cancer has been reported. Boosting the immune system response through exercise sessions can be beneficial in cancer patients (7). In hepatic steatosis and fatty liver disease, excessive accumulation of fat could be regulated by exercise workouts (2, 6). The increment and activation of AMP-activated protein kinases are processes which occur as part of the exercise mechanism for improving hepatic steatosis (6). Therefore, exercise could reduce the chance of liver metabolic syndromes by promoting metabolic pathways (8). In a proteomics study (2DGE coupled with MALDI-TOF MS/MS), it was suggested that exercise training could alter the proteome expression of aged rat liver by improving its metabolic function. Twelve proteins were identified as adaptive alterations in samples undergoing exercise treatment. Pathways of sulfur, glycolysis, and methionine metabolism were detected for these proteins in the original study (8). Proteomics can identify changes in protein expression that undergo environmental factor alterations (9). Bioinformatics, on the other hand, can provide a better understanding of biomarkers introduced by proteomics through studying them in a network pattern (10). In this way, it is possible to detect the most fundamental biomarkers and their crucial corresponding pathways in terms of centrality analysis. Consequently, these candidates can be selected for therapeutic interventions (9). 

The molecular mechanisms underlying exercise can be understood through post-analysis such as bioinformatics of proteomics data (11, 12). The health benefits of exercise for different parts of the human body have been extensively reported (13-15). One study clearly demonstrated that physical activity can postpone the aging of skeletal muscles (13). A panel of biomarkers in this regard could better describe the molecular mechanism of physical exercise. In view of that, proteomics data analysis of the liver proteome after physical activity was designated for bioinformatics evaluations. Hence, achieving new insights into the molecular mechanisms of exercise training on the liver can be reached by examining the bioinformatics of proteomics data of rat liver for therapeutic interventions. 

## Methods

**Data collection**

Proteomics study (2DGE) coupled with MALDI-TOF MS/MS was performed on liver tissue samples from rats. For this analysis, 16 aged male rats (*Rattus norvegicus*) samples, acquired from Guangdong Medical Laboratory Animal Center (GDMLAC), Guangdong, China, were divided into control and trained (8 CON and 8 EXE) groups. The rats underwent mediocre physical fitness to analyze this treatment on liver function (8). In this research, 12 differentially expressed proteins (DEPs) were identified by proteome analysis after rats underwent treadmill exercising sessions for 8 weeks. Half of the samples were shown to be up-regulated and half were down-regulated. 

**PPI network analysis**

Cytoscape 3.7.2 (https://cytoscape.org/) and its plug-ins constructed and analyzed a protein-protein interaction network of 12 candidates (16) using as a source String db v. 1.5.0 (http://string-db.org). Information retrieved from this database included disease name, compound, protein name, and PubMed. Protein name query was assigned for this research with the kappa score cut off of 0.5 (physical interaction weight). The weight ranged from 0-1, and the default setting was the score of 0.4 (17). The constructed network was then considered for centrality analysis with Network Analyzer. This application analysis degree (K) and betweenness centrality (BC) and the proteins with the highest values of the first ones were called hubs, and the second ones were called bottlenecks. Hub-bottlenecks are those nodes in the network that have both features (18). After assigning the central nodes, the ontology of the candidates was described with ClueGO v.2.5.6 and CluePedia v.1.5.6 (19, 20). The enrichment analysis designated for the nodes with the highest centrality was biological process characterizing. 

**Statistical analysis**

The statistical criteria for this analysis were three genes per term, a gene percentage in term of 4, and the corrected *p*-value was Bonferroni step down as the default setting. The kappa score cut off was set to 0.5. 

## Results

A network of 11 nodes and one link between two of them including PGM1 and ENC1 were concluded from the first network query of 12 differentially expressed proteins (DEPs) without the addition of any nodes. None of the nodes except two were in a direct connection (figure not shown).

After the addition of 50 neighbor nodes and considering the kappa score cutoff = 0.5, the second network with 61 and 667 links was obtained. 

**Figure 1:**
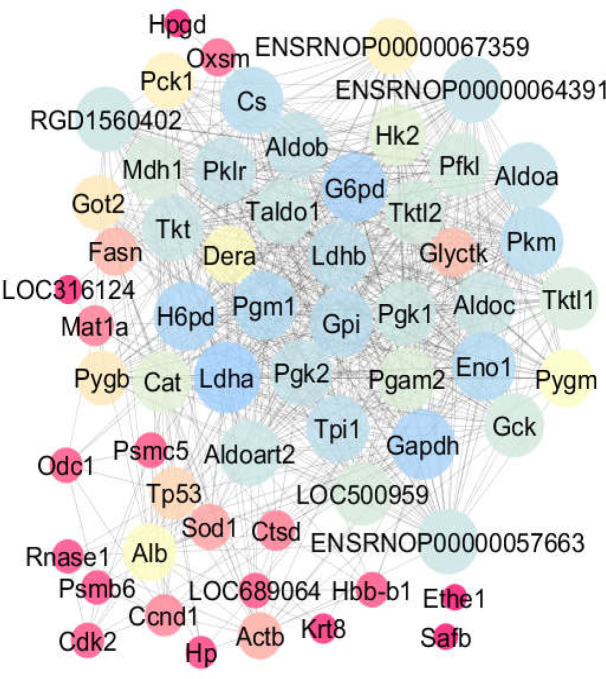
The second network (that including the additional genes) with confidence score cut off = 0.5 and addition of 50 nodes. Two nodes in the lower right corner are not linked from the query. The layout of the nodes is based on degree value

In the second network (shown in Figure 1), two nodes, namely Ethe1 and Safb, remained not connected in the map. 

To understand the topological features of the resulted network, Network Analyzer found the high degree (K) valued nodes as tabulated in Table 1. 

**Table 1:** Nodes are ordered based on K values. The 10% of highest values of degree are mentioned. ID of queried hubs is shown

R	Display Name	Query Term	K	BC
1	Eno1	P04764	35	0.01
2	Gpi		35	0.01
3	Pkm		35	0.04
4	Pgm1	P38652	34	0.03
5	Aldoa		33	0.02
6	Aldoart2		33	0.02

The high valued nodes in degree are called hubs; among them, two nodes, Eno1 and Pgm1, are from the queried ones. Enol1, Gpi, and Pkm had the highest degree, while Pkm showed the highest betweenness centrality. 

**Figure 2:**
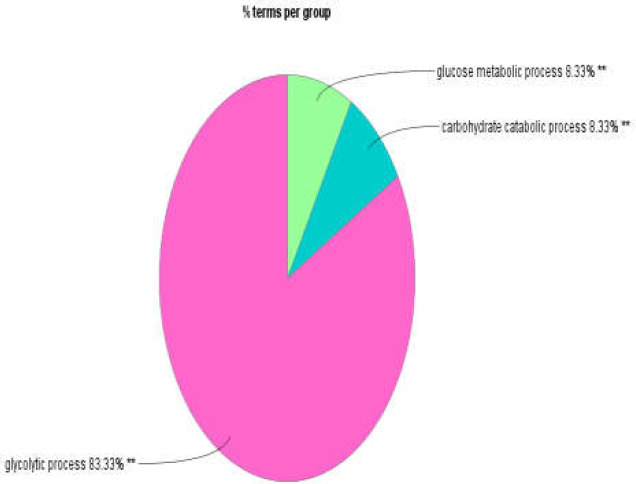
Pie chart view of biological processes of hubs showing three highlighted groups: glycolytic process, carbohydrate catabolic process, and glucose metabolic process. A p-value ≤ 0.01 was considered

To identify the biological properties of the retrieved hubs, enrichment analysis by ClueGO was done, and the results are shown in Figure 2. 

**Figure 3:**
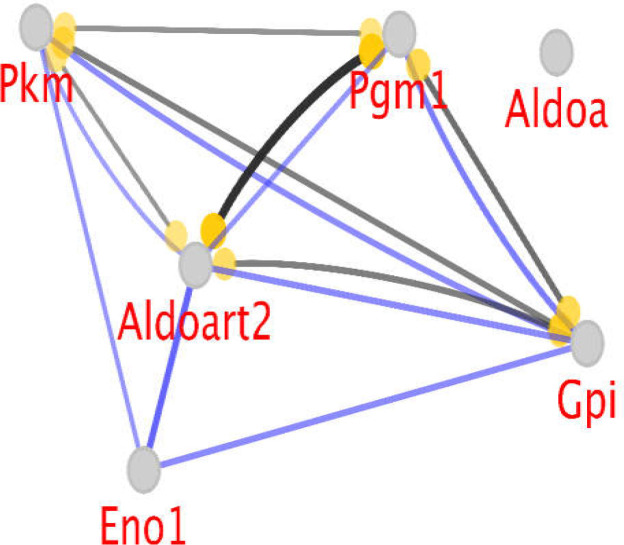
Action view between all hub genes. The black edge indicates reaction and the blue color edge shows binding correlation. Round tips refer to positive regulation

In Figure 2, the glycolytic process is the most highlighted BP group for the hub nodes. This term occupies the largest part of the pie. STRING action view in CluePedia demonstrates the notion of how hubs are functioning in Figure 3.

As edge score is shown by the thickness, those nodes with the highest and lowest interactions can be concluded from Figure 3. Aldoart2 and Pgm1 have the highest kappa score cut off of = 0.9, where the edge is very thick. Pgm1 and Gpi, which are ranked next in action, have a kappa score of 0.5. In addition, for the binding type action, Eno1 and Aldoart2 have the highest kappa score of = 0.6.

## Discussion

The molecular mechanism underlying exercise can be understood through post-analyses such as bioinformatics of proteomics data (11, 12). The health benefits of exercise for different parts of the human body have been extensively reported (13-15). For example, one study clearly demonstrated that physical activity can postpone skeletal muscle aging (13). A panel of biomarkers in this regard could better describe the molecular mechanism of physical exercise. In view of that, proteomics data analysis of the liver proteome after physical activity was designated for bioinformatics evaluations.

The protein-protein interaction network analyses of DEPs obtained from the proteomics including 12 differentially expressed proteins were evaluated to find those crucial to regulating body function after exercise. Not only the queried DEPs, but also the first neighbors were assessed. In the first step, Ethe1 and Safb were excluded from the other queried proteins due to the disability of both proteins in corporation in the network. Eno1 and Pgm1were highlighted among the queried proteins as the critical dysregulated proteins effected by exercise. Network analysis provided useful information, introducing Aldoart2, Aldoa, Gpi, and Pkm as the important related protein among the first neighbor proteins. The glycolytic process was emphasized as the significant class of biological processes that were provoked by exercise. Almost all the genes except Pgm1 contribute to the glycolytic process. Glucose and carbohydrate metabolism are the other two processes identified as relevant. Action map analysis revealed close relationships between the hub nodes except Aldoa. The finding is consistent with previous evidence for the outcome of exercise. 

Studies have shown that ENO1 as a glycolytic protein is also a cancer marker for malignancies such as head, neck, and gastric cancers (21, 22). In fact, glycolysis is the process through which cancer cells are provided their energy (23). This process, called the Warburg effect, is described as a common metabolic phenomenon in cancer progression. Since cancer cells divide promptly, they need an excessive intake of glucose (24). Moreover, inhibition of this process is a method of treatment in the cancer field (21). It has been shown that inhibition of ENO1 with shRNA inhibitor is an effective method for freezing the development of gastric cancer (22). ENO1 up-regulation is reported in some cancers (21, 22). In the proteomics study of the livers of trained rats, this protein is down-regulated. As one of the highest ranked down-regulated hub-bottlenecks of our network, ENO1 could be important in explaining the benefits of exercise training in diseases such as cancer, as its up-regulation is part of the cancer growth pathway. 

A literature review or other hubs that are not assigned as differentially expressed after training sessions in mice could be supportive for their feasible role in cancer progression. GPI as the second ranked protein in the hub category indicated some linkages to cancer. GPI transamidase (GPIT) and GPI anchored proteins are key markers of different types of cancers. GPIT and GPI have been described as promoting cancer growth (25, 26). In fact, subunits of the GPIT complex are dysregulated and show different expression patterns in ranges of cancers, which is dominantly overexpression. For example, GPI8 and GPAA1 are subunits of GPIT that demonstrate differential expression in liver cancer. Based on the evidence, expression changes in GPI8 and GPAA1 in liver cancer are down-regulated and up-regulated, respectively (26). 

The next protein characterized as a possible hub-bottleneck in the network is Pkm. As shown in Table 1, Pkm is the top bottleneck characterized with the highest betweenness centrality value. Overexpression of Pkm is highlighted as a prognostic feature in cancers such as tongue cancer (27). 

PGM1 is the fourth highlighted ranked protein that is over-expressed in trained rats. However, this protein shows down-regulation in hepatocellular carcinoma (HCC) as one of the most frequent types of liver cancer (28). This protein presents mostly in high demanding metabolism tissues of the liver and muscles (24). PGM1 regulates the glycogenesis process, the dysregulation of which affects glucose catalysis and, consequently, cancer progression (28). Thus, as mentioned above, trained subjects may show modulation of this protein in comparison with normal cases, and that could be important in cancer treatment. Metabolism in cancer cells differs from normal cells, and cancer development depends highly on glucose consumption (28), which, according to the current findings, could apparently be regulated by physical activity. 

Evidence exists about the role of varieties of aldolase molecules in cancer promotion. Researchers have reported dysregulation of aldolase in lung and colon cancers (29, 30). Aldolase-a and aldolase-art2 are assigned as two key hubs in the current investigation; however, aldolase-a was isolated in the action map, as shown in Figure 3. 

As indicated earlier, all of the hubs are interactive in the glycolytic process except for Pgm1 as one of the differentially expressed proteins. This involvement shows their possible importance in cancer development and the potential regulatory effects of physical activity. In the other words, exercise may also affect the other dysregulated proteins that are not reported by the original proteomics study (8). It seems that in addition to the commonly known benefits of physical activity, cancer prevention and anti-cancer properties could be other significant features of exercise. Therefore, a complementary study in this regard could be beneficial; however, diversity in research methods are limitations of investigations. It is suggested that unique styles and patterns of exercise be considered in future assessments. 

This study suggests the potential role of metabolic hubs as therapeutic targets in liver-related diseases, especially cancer, after complementary investigations. It is suggested that the evaluation of the nominated proteins be analyzed in patients with malignancies undergoing physical activities.

## Conflict of interests

The authors declare that they have no conflict of interest.
